# Successful sequential therapy with rituximab and telitacicept in refractory Anti-NMDA receptor encephalitis and MOG-associated demyelination: a case report and literature review

**DOI:** 10.3389/fimmu.2025.1509143

**Published:** 2025-02-06

**Authors:** Jingliang Zhang, Minzhe Hu, Chunjuan Wang, Shougang Guo

**Affiliations:** Department of Neurology, Shandong Provincial Hospital Affiliated with Shandong First Medical University, Jinan, Shandong, China

**Keywords:** NMDAR encephalitis (NMDARE), telitacicept, rituximab, treatment, MOGAD

## Abstract

Clinical management of the rare and complex overlapping syndrome of MOG-antibody disease and anti-NMDAR encephalitis (MNOS), which has an uncertain pathogenesis and a high risk of recurrence, is highly challenging. We describe the case of a 19 years-old female patient, who first complained of headache, fever, and irritability. After that, she experienced frequent seizures and mood disorders. The diagnosis of MNOS was verified through antibody tests and imaging. For the patient, intravenous immunoglobulin and high-dose methylprednisolone were effective as first-line immunotherapy. Long-term immunotherapy with oral prednisone and mycophenolate mofetil was used to prevent relapses. However, over six years, the patient had five relapses when the mycophenolate mofetil dosage was reduced. The patient’s condition stabilized after taking rituximab as second-line immunotherapy, with less than 1% of total lymphocytes being CD19+ cells. Eleven months later, the plasmablast ratio increased, and patients experienced new symptoms such as bilateral optic neuritis. After that, the patient got telitacicept injections regularly for 13 months, during which time her symptoms subsided, and there were no adverse effects or relapses. This case suggests that telitacicept may be a viable adjunct or sequential therapy option for the depletion of B cells in MNOS.

## Introduction

Myelin oligodendrocyte glycoprotein antibody-associated disease (MOGAD) is an immune-mediated inflammatory demyelinating disorder of the central nervous system, recently found and defined by the presence of anti-MOG antibodies (1). Autoimmune encephalitis (AE) is a set of illnesses associated with antibodies against neuroglial antigens. The most common AE association is anti-NMDAR encephalitis, which accounts for around 54% to 80% of all AE cases (2). Previously, MOGAD and anti-NMDAR encephalitis were thought to be two independent illnesses, each associated with a different specific pathogenic mechanism. However, new researches revealed that these two disorders can coexist (3–6), leading to overlapping clinical symptoms. Currently, researchers classify patients with clinical and imaging features in common between the two diseases and coexistence of both anti-MOG and anti-NMDAR antibodies as having MOGAD and NMDAR encephalitis overlap syndrome (MNOS) (6).

MNOS is characterized by frequent relapses, and no standardized treatment protocol is available. In the acute phase, treatment of MNOS generally includes intravenous methylprednisolone (IVMP), intravenous immunoglobulin (IVIG), or plasmapheresis (PE). Long-term treatments like oral prednisone, mycophenolate mofetil (MMF), rituximab (RTX), and azathioprine (AZA) can be given to some individuals to avoid relapse following acute treatment (4, 6–9). RTX, an anti-CD20 chimeric monoclonal antibody, targets and depletes CD20-positive B cells, including initial, mature, and memory B cells. In addition, telitacicept, a novel recombinant fusion protein that is composed of the human IgG Fc component and the ligand-binding domain of the TACI receptor, neutralizes two critical cell signaling molecules, B lymphocyte stimulator (BLyS) and proliferation-inducing ligand (APRIL) (10). This action impedes the development and survival of plasma and mature B cells. To date, telitacicept has been proven to be effective as treatment strategy in neuromylitis optica spectrum disorder (NMOSD), Myasthenia gravis (MG), and other neuroimmunological conditions (11, 12). Here, we present a case of refractory MNOS where rituximab and telitacicept were used sequentially to limit clinical relapse successfully.

## Case presentation

**Figure 1:**
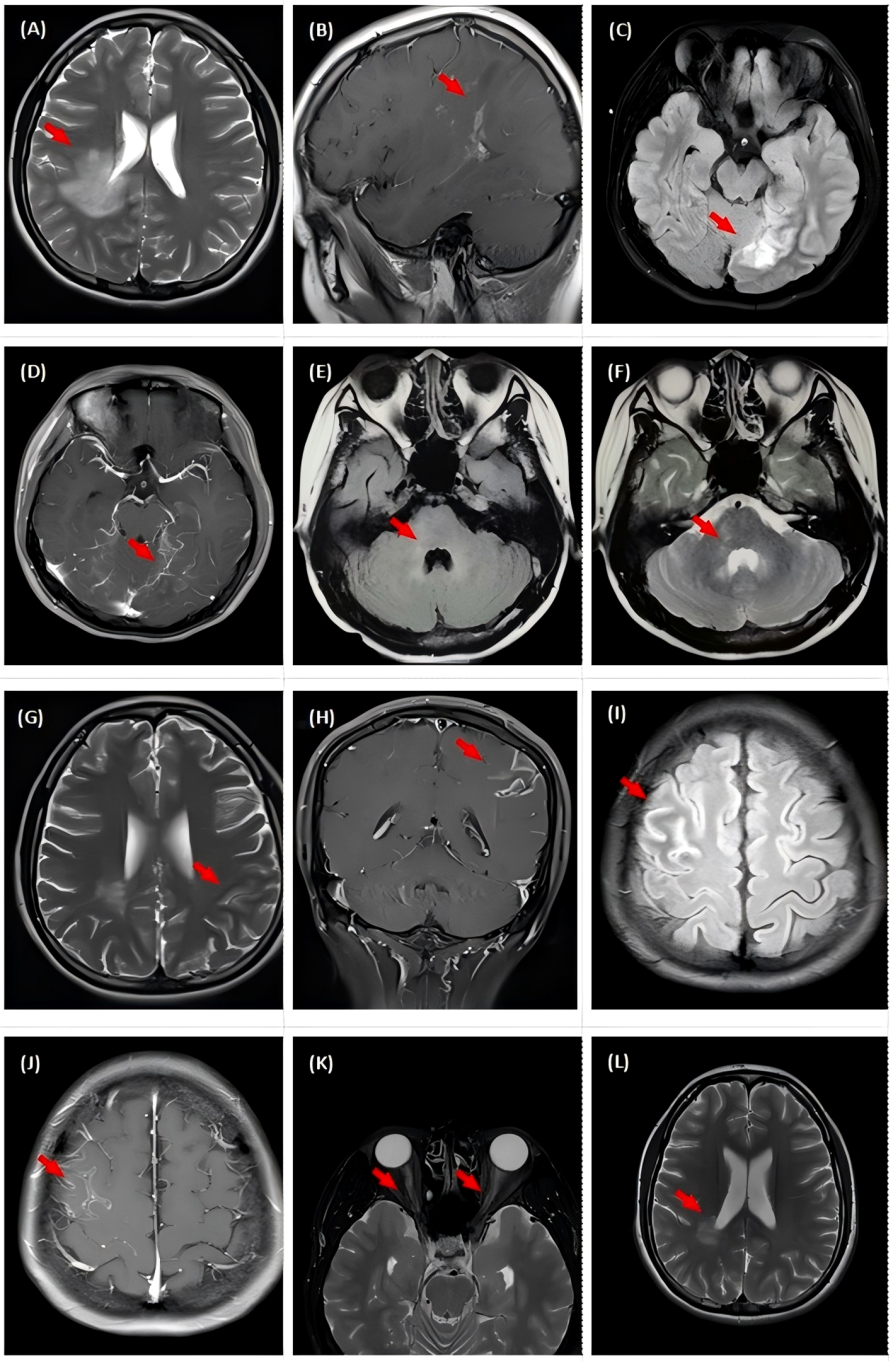
Magnetic Resonance Imaging of the brain and spinal cord. **(A, B)** Brain MRI at onset showing abnormal signals on the right corona radiata and basal ganglia regions. **(C, D)** Brain MRI showing alterations on left temporal an occipital lobes with enhancement on FLAIR and T1 post-Gd during second attack. **(E, F)** Abnormal signal on T2/FLAIR in the right pons at the time of the patient’s third attack. **(G, H)** New lesion in the left parietal lobe with contrast-enhancement during fourth attack. **(I, J)** New lesions in the right frontal cortex with linear contrast-enhancement during fifth attack. **(K)** Abnormal signal in both optic nerves, following treatment with RTX at the time of the patient’s sixth attack. **(L)** Lesions after 1 year of treatment with telitacicept.

A 19-year-old female was admitted to the hospital in September 2016, presenting with headache, fever (up to 39°C), and irritability. The patient did not report any joint discomfort or any anomalies in the muscles or skin, and a physical examination, which included a neurological evaluation, revealed no significant results. Routine blood exams, including immunological tests, creatine kinase (CK) levels, rheumatoid factor, and antistreptolysin O, were all within normal limits, exlcuding systemic autoimmune rheumatic conditions. The cerebrospinal fluid (CSF) pressure was 190 mmH_2_O, and the examination showed increased leucocytes (130/mm³, 69% mononuclear) and normal protein levels (0.37g/L, normal range <0.4g/L). Polymerase chain reaction (PCR) analysis for Mycobacterium tuberculosis, herpes simplex virus (HSV), cytomegalovirus (CMV), Epstein-Barr virus (EBV), and cryptococcal DNA yielded negative results. The magnetic resonance imaging (MRI) revealed a hyperintense lesion in the right splenium of the corpus callosum, corona radiata, and basal ganglia, as observed on fluid-attenuated inversion recovery (FLAIR) imaging (**Figure 1**). These findings demonstrated that the patient met the diagnostic criteria for encephalitis of presumed autoimmune etiology established by the International Encephalitis Alliance in 2013 (13). She received acyclovir and methylprednisolone (500 mg/d for 3 days and 240 mg/d for 3 days), resulting in a swift improvement in symptoms. A subsequent CSF examination one month later revealed a decrease in pressure (178 mmH_2_O) and leucocyte count (24/mm³, 100% mononuclear). After discharge, the patient was prescribed 60 mg/day oral prednisone, which was methodically tapered until stop over three months.

In May 2018, the patient was referred to our hospital due to recurrent headaches and fever. During hospitalization, she experienced daily recurrent generalized tonic-clonal seizures (GTCS). There were no noticeable abnormalities on the neurologic exam. Brain MRI scanning showed a new lesion in the left temporal and occipital lobes. The 24-hour video electroencephalogram (VEEG) identified an aberrant background rhythm marked by diffuse paroxysmal discharges within the theta frequency band. CMV DNA, EBV DNA, and varicella-zoster virus (VZV) nucleic acid were not detected in serum or CSF by PCR, and antibodies to HSV types I and II, EBV, CMV, and rubella virus were also negative by ELISA. Immunologic tests, such as antinuclear antibodies, antineutrophil cytoplasmic antibodies, and anticardiolipin antibodies (via ELISA), showed no abnormalities. In addition, we employed a commercially available immunoblotting assay (Euroimmun, Lübeck, Germany) to detect Ri, Yo, Hu, Ma2, Amphiphysin, and CV2 IgG in serum, and the results were likewise negative. Moreover, serum tests for anti-aquaporin-4 (AQP4), anti-myelin basic protein (MBP), anti-NMDAR, anti-MOG, anti-LGI1, anti-CASPR-2, anti-AMPA R1/R2, and anti-GABABR conducted via fixed cell-based assay (CBA) by Hangzhou Dian Medical Laboratory Center yielded negative results. The patient was treated with 500 mg of IVMP for five days with subsequent tapering (240 mg/d for three days and 120 mg/d for three days), and intravenous acyclovir. Her clinical symptoms improved following the treatment, and she was discharged from the hospital with a prescription for oral prednisone at a daily dose of 60 mg, followed by tapering.

In July 2018, following the reduction of the oral prednisone dose, the patient exhibited a flare-up of symptoms characterized by intermittent fever, mood instability, and unsteady gait. She was later referred to Peking Union Medical College Hospital, where neurological examination indicated left-sided limbs weakness, ataxia, and bilateral Babinski sign. The brain MRI showed a new lesion involving the right pons (**Figure 1**). CSF examination revealed normal pressure, white blood cell count, and protein levels. Laboratory tests identified positive MOG IgG in serum and NMDAR IgG in CSF, with titers of 1:100 and 1:10, respectively, as assessed by fixed CBA from Neurology Pathology Laboratory of Peking Union Medical College Hospital. Based on the patient’s clinical symptoms, antibody test results, and MRI, the patient meets the diagnostic criteria for both MOGAD and anti-NMDA receptor encephalitis (1, 14). Eventually, diagnosis of MNOS was made, according to the concept of Fan et al. (6). The patient received IVMP, initiating with 1,000 mg daily for 3 days, subsequently reducing to 500 mg daily for 3 days, and tapering to 250 mg and 120 mg daily over the following 6 days. Immunoglobulin was given at a dosage of 0.4 g/kg daily for 5 days after IVMP, alongside oral MMF at 0.5 g administered twice daily. This treatment led to clinical enhancement. Upon discharge, the patient was treated with oral prednisone at a dosage of 60 mg per day, gradually decreasing by 5 mg every one to two weeks, and MMF at 1 g per day.

**Figure 2:**
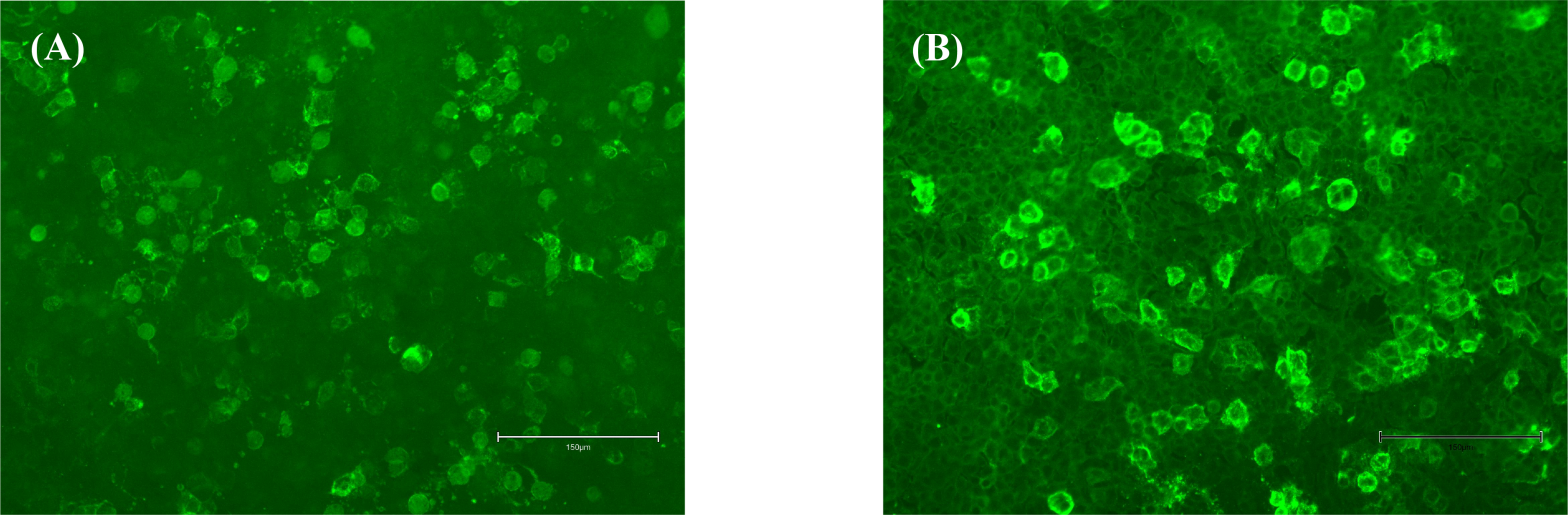
The antibody test results of serum and cerebrospinal fluid (CSF) during the patient’s fourth relapse. **(A)** CSF showing the binding to the surface of the cells expressing anti-NMDAR receptors (NMDAR) (titer 1:1). **(B)** Myelin oligodendrocyte glycoprotein (MOG) antibodies (titer 1:10) were detected in the serum.

**Figure 3:**
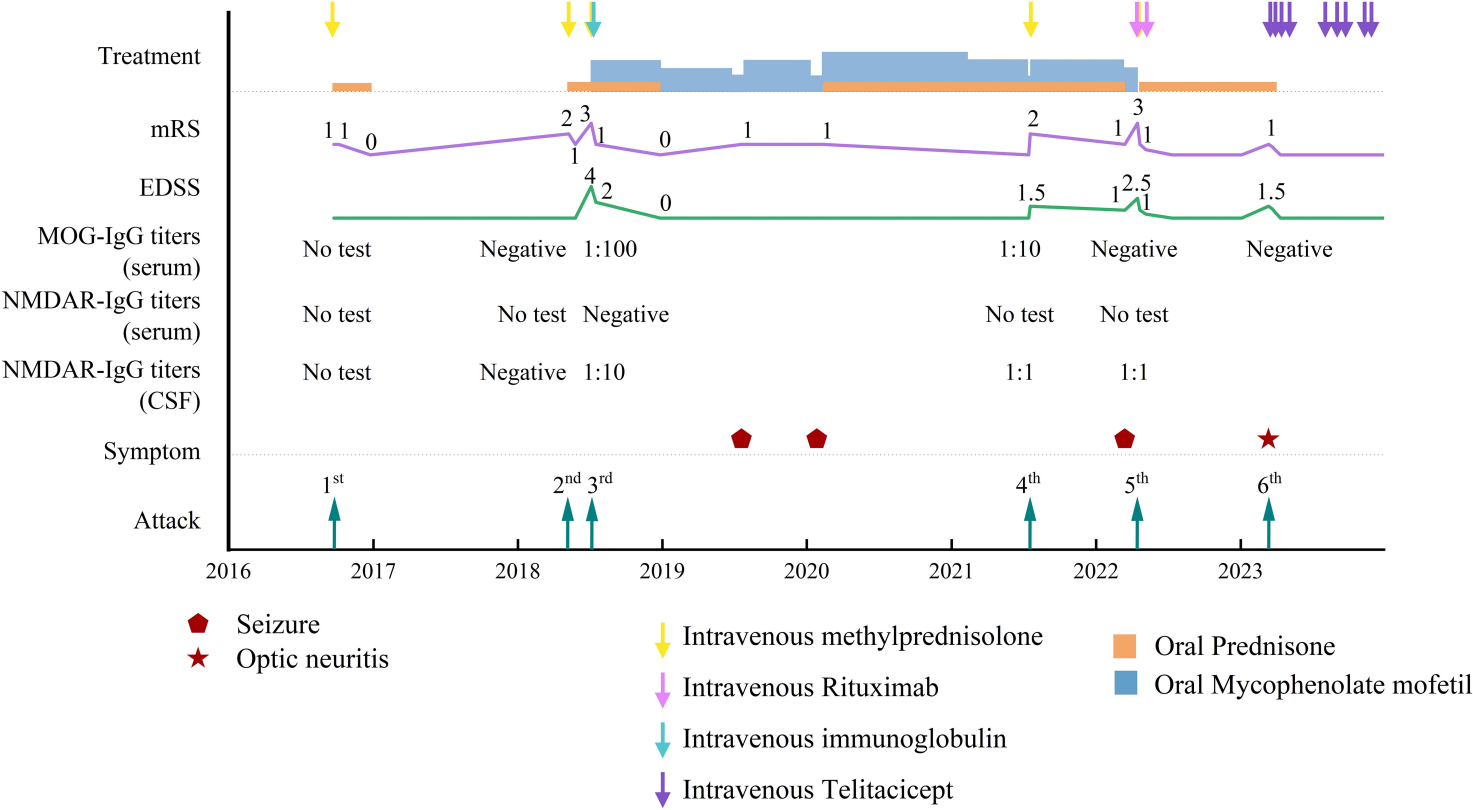
Temporal variation of symptoms, MOG-IgG titers, NMDAR-IgG titers, EDSS, and mRS in relation with treatment status.

In January 2019, the patient exhibited no additional aberrant symptoms and then discontinued prednisone and MMF was reduced to 0.75 g daily. After six months, MMF was further reduced to 0.5 g/day, at which point a new generalized motor seizure occurred. Increasing the MMF dose to 1 g/day resulted in no more seizures. The brain MRI revealed no new or enhanced lesions. In the subsequent two years, the patient experienced two analogous episodes, both coinciding with decreases in the MMF dosage. In 2021 and the initial quarter of 2022, the patient had two relapses after reduction in MMF dosage, with each episode linked to new lesions on the left parietal lobe and right frontal cortical regions (**Figure 1**). During the patient’s fifth attack, the patient experienced seizures again, and FLAIR showed new lesions on the right frontal cortical area, which were termed as FLAIR-hyperintense lesions in anti-MOG-associated encephalitis with seizures (FLAMES). Fixed CBAs for serum MOG IgG and CSF NMDAR IgG showed low positive results at titers of 1:10 and 1:1, respectively (**Figure 2**). After counseling with the patient, rituximab (RTX) treatment was initiated in April 2022, with a 500 mg infusion, followed by an additional 500 mg infusion two weeks later. After eleven months, the patient experienced bilateral optic neuritis. The peripheral blood CD19+/lymphocyte ratio was notably low, measuring only 0.06%, far below the threshold required for RTX re-treatment. However, the proportion of memory B lymphocytes and plasmablasts had increased to 4.15% and 0.46%, respectively. In March, 2023, the patient began treatment with telitacicept, initially at the dose of 160 mg/week for the first three months, and after repeated every 2-4 weeks. During the subsequent 16 months, the patient exhibited no additional relapses and reported no notable discomfort or adverse effects. The patient has been off prednisone for six months and has returned to normal daily activities. **Figure 3** illustrates the disease progression over the past eight years.

## Review of the literature

A literature review was conducted using PubMed, and 11 case reports and studies were identified regarding telitacicept in treating neuroimmune diseases (see **Table 1**). The studies demonstrated the notable efficacy of telitacicept in multiple autoimmune conditions. Six of the studies reviewed examined its use in MG, two in NMOSD, one in idiopathic inflammatory myopathy (IIM), one in autoimmune neuropathies (AN), and one in systemic lupus erythematosus (SLE) combined with MOGAD. In these instances, telitacicept was predominantly employed as an add-on or sequential therapy as a result of inadequate responses to prior treatments (Studies 3, 10, 11), notable adverse effects (Studies 1, 2, 3, 8, 9, 10, 11), or relapses after prolonged use of other treatments (Studies 1, 6, 7, 8, 9). Telitacicept exhibited significant efficacy, irrespective of its use as a monotherapy or in combination with other immunosuppressive agents. In the six MG studies, telitacicept facilitated disease stabilization, evidenced by enhancement in the Quantitative Myasthenia Gravis Score (QMG) and additional clinical scoring systems. The two NMOSD studies (1 and 8) demonstrated that telitacicept decreased disease relapses and improved symptoms. Several studies (2, 6, 7, 9, 11) found that telitacicept facilitated a reduction in the dosage of steroids and other immunosuppressants, consequently minimizing associated side effects. In patients with IIM, MOGAD, AN, telitacicept similarly resulted in significant clinical improvement and prolonged relapse intervals.

**Table 1:** Literature review of studies on telitacicept use in neuroimmune disorders.

	Study (year)	Study design	Diagnosis	Antibody testing	Primary immunotherapy	Reason for use	Immunotherapy	Response to treatment
1	Ding et al. (2022) (12)	Research article	NMOSD	AQP4-ab	AZA, PDN, MMF	Relapse or adverse effects	Plasma exchange, followed by telitacicept (240 mg/qw) for 46 times	2 patients relapse, 5 patients remained relapse free after 48 weeks of treatment. The time to first recurrence was prolonged and the number of recurrences was reduced.
2	Guo et al. (2023) (15)	Case reports	MG	AchR-ab, Titin-ab, RyR-ab	PDN, IVIG, TAC	Advanced age, in order to reduce the use of steroids and TAC.	PDN, tacrolimus, add-on telitacicept (160mg/qw) for 3 months	The steroid dosage, QMG score, and antibody titers all decreased. At the last follow-up (7 months), there was no relapse, and prednisone had been discontinued for 3 months
3	Ren et al. (2023) (16)	Case reports	AN	NF155-ab	PE, MMF, RTX, IA, PDN	Severe adverse effects, inadequate response, and elevated antibody titers.	Telitacicept (160mg/qw) for 4 times.	At the last follow-up, steroids were discontinued, and both INCAT scores and antibody titers had decreased
4	Tian et al. (2023) (17)	Case Reports	SLE MOGAD	MOG-ab,dsDNA-ab	NM	NM	IVMP, MMF, HCQ, telitacicept (160mg/qw)	symptoms disappeared after a week, ESR, anti-dsDNA and C3/C4 declined, brain and full spine MRI improvement
5	Yin et al. (2023) (18).	Research article	MG	AchR-ab,MuSK-ab	NM	NM	TAC, AZA, MMF, add-on telitacicept (240mg/160mg/qw)	After 24 weeks, the patient’s symptoms stabilized, with a decrease in QMG scores, B cells, IgA, IgG, IgM, and other markers.
6	Zhang et al. (2024) (19)	Case Reports	MG	AchR-ab, RyR-ab	TAC, RTX, MMF, AZA, CsA	Relapse	PDN, telitacicept (160mg/qw) followed by efgartigimod (800 mg/qw)	The QMG score decreased, and the dosages of steroids and pyridostigmine were reduced, there were no relapses after 8 months of treatment.
7	Lin et al. (2024) (20)	Research article	MG	AchR-ab, Titin-ab, MuSK-ab	AZA,TAC, PDN, IVIG, PE, MMF, MTX, IL-6R, CTX, RTX	Relapse	PDN, add-on Telitacicept (160 mg qw/q2w)	At the observation endpoint (6 months), the QMG scores, MGSTI scores, and MG-ADL scores of 10 patients all significantly improved, and the steroid dosage was reduced.
8	Li et al. (2024) (21)	Case reports	NMOSD	AQP4-ab,anti-Ro-52-ab, anti-mitochondrial M2-ab, ANA	IVMP, MMF, IVIG, CTX	adverse effects, relapse	PDN,Telitacicept (160 mg/qw), followed by inebilizumab (300 mg, administered intravenously on days 1 and 15)	At the last follow-up (14 months later), the patient’s condition was stable, and the steroid dosage had decreased.
9	Zhang et al. (2024) (11)	Case reports	MG	AchR-ab	TAC, PDN, PLEX, IVIG	adverse effects, relapse	PDN, telitacicept (160mg/qw)	Two patients showed a steroid reduction and improvement in QMG and MG-ADL scores at the last follow-up.
10	Gao et al. (2024) (22)	Research article	IIM	NM	PDN, MTX, TAC, MMF, CTX, IVIG, PE	adverse effects, inadequate response	PDN, Add-on telitacicept (160mg/qw)	All patients showed a significant reduction in steroid dosage, improvement in MMT-8 scores, and a decrease in CK levels.
11	Wang et al. (2024) (23)	Case reports	MG	AchR-ab,MuSK-ab	TAC, PLEX, PDN, MMF, efgartigimod	adverse effects, inadequate response	PDN, MMF, RTX(375 mg/m^2^/6 months), telitacicept (160mg/qw)	Symptoms continued to improve, with a reduction in QMG scores, ADL scores, and dosages of steroids and MMF.

AChR-Ab, anti-acetylcholine receptor antibody; AN, autoimmune nodopathies; ANA, antinuclear antibodies; AZA, azathioprine; CK, creatine kinase; CsA, CyclosporineA; CTX, cyclophosphamide; HCQ, Hydroxychloroquine; IIM, idiopathic inflammatory myopathy; IA, Immunoadsorption; IL-6R, interleukin-6 receptor antagonists; INCAT, Inflammatory Neuropathy Cause and Treatment; IVIG, intravenous immunoglobulin; IVMP, intravenous methylprednisolone; MG-ADL, myasthenia gravis activity of daily living; MGSTI, myasthenia gravis treatment status and intensity; MuSK-Ab, muscle-specific tyrosine kinase antibody; MMF, mycophenolate mofetil; MOGAD, Myelin Oligodendrocyte Glycoprotein Antibody Disease; MTX, methotrexate; NM, not mentioned; NMOSD, neuromyelitis optica spectrum disorders; PDN, prednisone; PE, plasmapheresis; PLEX, plasma exchange; QMG, quantitative myasthenia gravis; RTX, Rituximab; RyR, Ryanodine receptor; SLE, systemic lupus erythematosus; TAC, Tacrolimus.

## Discussion

We present the first report of sequential treatment with rituximab and telitacicept in a patient with refractory MNOS. Titulaer’s initial report on MOGAD cases associated with anti-NMDA receptor encephalitis (8) led to a significant rise in related publications. In 2018, Fan et al. presented the concept of MNOS (6). After that, Hikari suggested that the presence of anti-NMDAR and anti-MOG antibodies in autoimmune encephalitis is a new clinical entity (24).

As understanding of this syndrome continues to evolve, universally accepted diagnostic criteria have yet to be established. Nevertheless, many case reports and clinical studies indicate that antibody positivity should be correlated with the clinical phenotype. Without corresponding clinical manifestations, diagnosing of the antibody-associated disease is not feasible, and the presence of antibodies should be regarded as an unspecific association. Several studies have found that when both anti-NMDA receptor and anti-MOG antibodies are present, the clinical symptoms are predominantly those of anti-NMDA receptor encephalitis (9, 24–26). These include cognitive deficits, psychosis, seizures, movement disorders, and so forth (27). Syndromes typical for MOGAD, including optic neuritis, myelitis, and acute disseminated encephalomyelitis, are relatively uncommon. The patient described in our report had several relapses, with lesions affecting the thalamus, pons, centrum semiovale, and cerebral cortex (**Figure 1**). Based on clinical and radiological characteristics, the patient sequentially exhibited features of anti-NMDAR encephalitis, FLAMES, and optic neuritis, which are consistent with the coexistence of anti-NMDAR encephalitis and MOGAD(conformed to the concept of MNOS by Fan (6)).

In the acute phase of MNOS, glucocorticoids, IVIG, and PE are frequently employed. Ding et al. reported a recurrence rate of 63.4% for MNOS, with 50.0% of patients experiencing relapses as encephalitis and 53.8% presenting as demyelinating events (28). A systematic review and meta-analysis involving 43 patients with dual positivity for MOG and NMDAR antibodies found that 15 of these patients experienced relapses (9). The findings indicate that MNOS exhibits a significantly elevated relapse rate. Given this, it is imperative to implement long-term immunotherapy in order to prevent relapses. The immunosuppressants utilized for long-term management correspond with the treatment strategies for anti-NMDAR encephalitis and MOGAD, such as prednisone, MMF, AZA, CTX, and RTX (3, 6, 29–33). In some patients with recurrent relapses, symptoms have been managed through the use of other immunosuppressants as a bridge. Of note, there are currently no reports of relapses in MNOS after treatment with rituximab.

**Figure 4:**
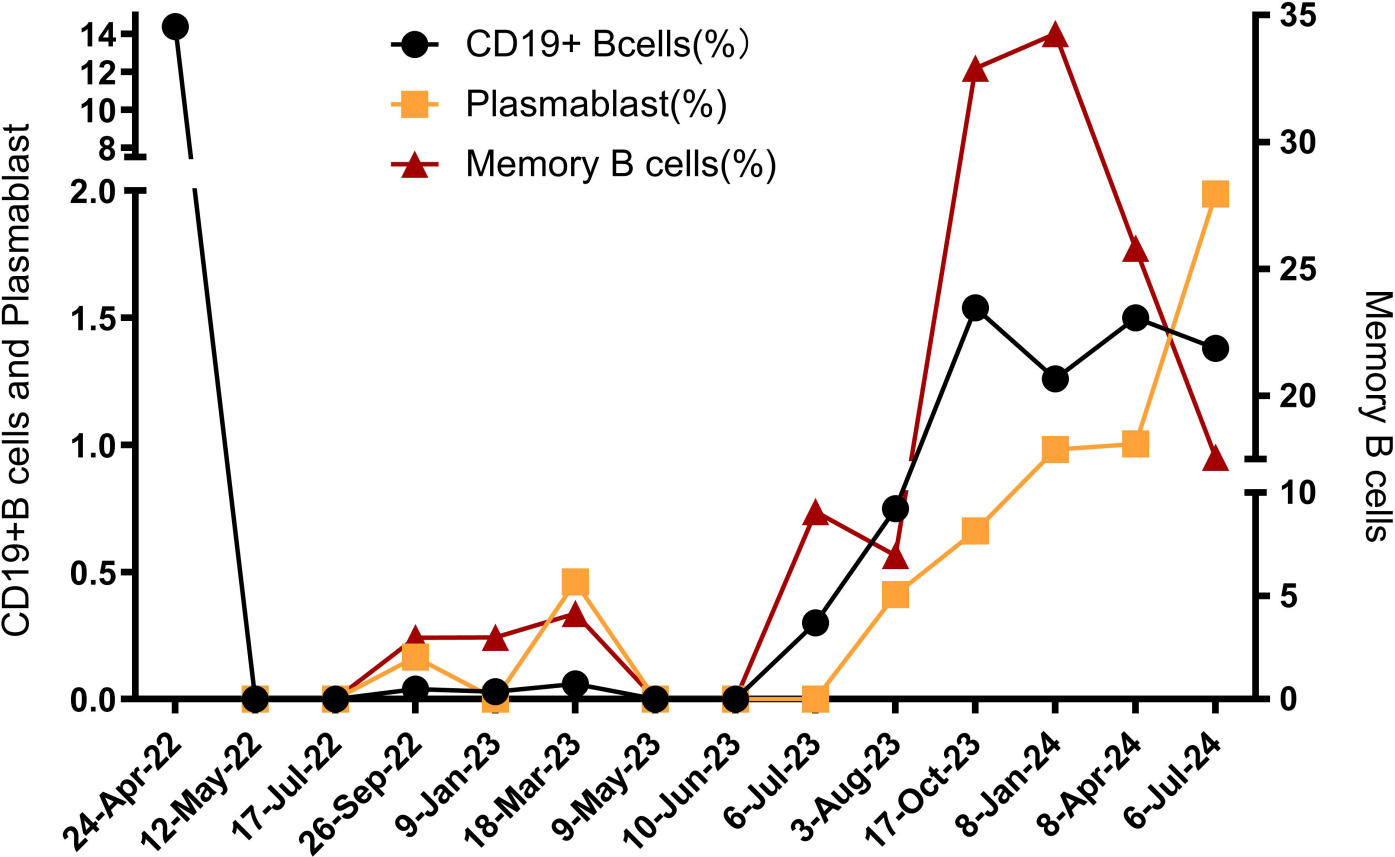
The percentage of CD19+B cells, plasmablast, memory B cells during treatment with RTX and telitacicept.

MMF inhibits T and B lymphocyte proliferation by inhibiting inosine monophosphate dehydrogenase (34). This patient received MMF treatment in 2018. However, during drug tapering, relapses and symptom variations led us to switch to RTX. RTX depletes CD20-positive B cells through different mechanisms including antibody-dependent cellular cytotoxicity (ADCC) and complement-dependent cytotoxicity (CDC) (35, 36). During the eleven months after RTX administration, CD19 was utilized as a marker to evaluate B cell depletion (37). Despite low CD19+ B cells levels, the patient experienced bilateral optic neuritis, as evidenced by brain MRI (**Figure 1**). Later, she presented with headaches and blurred vision. A brain MRI scan revealed bilateral optic neuritis (**Figure 1**). At this point, serum anti-MOG IgG was tested and found to be negative, with a CD19-positive cell proportion of 0.06%. This implies that evaluating relapse risk exclusively using CD19+ B cells need to be more thorough and accurate (**Figure 4**). Future research is required to identify more precise biomarkers for monitoring relapse rates in B cell depletion therapy for neuroimmunological diseases.

Multiple factors may contribute to the persistent relapse recurrency of neuroimmune diseases despite use only RTX. Firstly, RTX is capable of eliminating short-lived plasma cells (SLPCs). However, it is ineffective in the eliminating of long-lived plasma cells (LLPCs) or plasmablasts in the bone marrow, as these cells lack CD20 expression (38–40). LLPCs can migrate to the bone marrow as antibody-secreting cells (ASCs). These cells can then proliferate and differentiate into short-lived plasma cells, extending antibody secretion (16). Secondly, repetitive RTX administration may reactivate specific autoreactive memory B cells resistant to RTX, enhancing production of pathogenic plasma cells and activating immune responses (41). Thirdly, patients who undergo multiple RTX treatments may develop anti-RTX antibodies, which can neutralize RTX activity, leading to a higher relapse rate and quicker B cell reconstitution (42, 43). Fourth, polymorphisms in the FCGR3A gene can influence the efficacy of RTX in B cells depletion, with the quantity of FCGR3A-158V allele copies correlating with RTX-induced ADCC (44). This study observed that the patient received only two RTX injections. Although the proportion of CD19+/lymphocytes remained low, there was a gradual increase in the proportion of memory B cells and plasmablasts. Thus, the patient’s higher relapse rate may be associated with the persistence of LLPCs, memory B cells, or the FCGR3A-158V variant allele copies.

BLyS and APRIL are essential cytokines for the growth and development of B cells and plasma cells (45). Studies have demonstrated that BLyS and APRIL levels are significantly elevated in peripheral blood and CSF during the acute phases of NMOSD and anti-NMDAR encephalitis. These levels correlate with Expanded Disability Status Scale (EDSS) scores and disease prognosis (12, 46, 47). Moreover, BLyS levels increase notably in patients receiving multiple RTX infusions (48, 49). Despite consistently low CD19+/lymphocyte levels, the patient displayed increased proportions of plasmablasts, indicating heightened disease activity. Similar results have been reported in systemic lupus erythematosus (SLE) and IgG4-related diseases, emphasizing the potential of plasmablast surveillance to evaluate disease activity, which may be linked to BLyS and APRIL levels (50–53). Telitacicept inhibits the development and survival of plasmablasts and mature B cells by neutralizing BLyS and APRIL (10). Guan et al. described treating eight patients with refractory NMOSD utilizing telitacicept sequential with plasma exchange. Among them, five (63%) did not relapse within 48 weeks, while two patients (25%) experienced relapses at 45 and 234 days post-treatment, respectively. The interval between relapses was significantly extended, and the overall relapse frequency decreased markedly compared to pre-treatment levels (P < 0.001). Non-relapsed patients also showed a mean EDSS score reduction of 0.83 points (range 0.5–2) (54). Considering the evidence presented, telitacicept was chosen as a bridging therapy alongside RTX for our case.

The patient’s response to telitacicept was remarkable, with the EDSS and the modified Rankin Scale (mRS) scores dropping to 0 one month after initiating telitacicept (**Figure 2**). The observed clinical improvement correlated with a notable decrease in CD19+ B lymphocytes, plasmablasts, and memory B cells, all falling below the detection threshold. At the most recent follow-up, 16 months following the regular administration of telitacicept, the percentage of CD19+ B cells increased slightly to above 1%, while the proportion of plasmablasts and memory B cells remained low (**Figure 4**).

Our study has several limitations. First of all, the study is a case report with a small sample size; additional research with more cases is required to confirm the viability of telitacicept and rituximab treatment in succession. Additionally, extended follow-up is required to assess this treatment method’s long-term efficacy and safety. Furthermore, this study did not evaluate changes in BLyS or APRIL levels in serum or CSF, which could yield important insights into the mechanism of action. Also, we did not test the serum and CSF using fixed tissue-based assay (TBA). Despite these limitations, our study offers a promising new treatment option for patients with refractory MNOS and provides a potential bridging therapy for B cell depletion in neuroimmunological diseases. The potential of telitacicept to substitute traditional B cell depletion therapies or its role in conjunction with other immunotherapies for enhanced efficacy is a critical issue that necessitates further research.

## Conclusion

This exploration presents a clinically significant opportunity for refractory MNOS patients, potentially reducing medical costs and minimizing drug-related side effects. The safety and efficacy of this therapy will be further evaluated to extend its application to other neuroimmune diseases, including autoimmune encephalitis and NMOSD, through the implementation of exploratory case series or clinical investigations.

## Data Availability

The original contributions presented in the study are included in the article/supplementary material. Further inquiries can be directed to the corresponding authors.
